# RETRACTION: Effectiveness of Concentrated Growth Factor and Laser Therapy on Wound Healing, Inferior Alveolar Nerve Injury and Periodontal Bone Defects Post‐Mandibular Impacted Wisdom Tooth Extraction: A Randomized Clinical Trial

**DOI:** 10.1111/iwj.70528

**Published:** 2025-04-18

## Abstract

**RETRACTION:** LuZ. , BingquanH. , JunT. , and FeiG. , “Effectiveness of Concentrated Growth Factor and Laser Therapy on Wound Healing, Inferior Alveolar Nerve Injury and Periodontal Bone Defects Post‐Mandibular Impacted Wisdom Tooth Extraction: A Randomized Clinical Trial,” International Wound Journal 21, no. 1 (2024): e14651, 10.1111/iwj.14651.38272792 PMC10789919

The above article, published online on 15 January 2024, in Wiley Online Library (http://onlinelibrary.wiley.com/), has been retracted by agreement between the journal Editor in Chief, Professor Keith Harding; and John Wiley & Sons Ltd. Following an investigation by the publisher, all parties have concluded that this article was accepted solely on the basis of a compromised peer review process. The editors have therefore decided to retract the article. The authors did not respond to our notice regarding the retraction.

**RETRACTION:** Z. Lu , H. Bingquan , T. Jun , and G. Fei , “Effectiveness of Concentrated Growth Factor and Laser Therapy on Wound Healing, Inferior Alveolar Nerve Injury and Periodontal Bone Defects Post‐Mandibular Impacted Wisdom Tooth Extraction: A Randomized Clinical Trial,” International Wound Journal 21, no. 1 (2024): e14651, 10.1111/iwj.14651.38272792 PMC10789919

The above article, published online on 15 January 2024, in Wiley Online Library (http://onlinelibrary.wiley.com/), has been retracted by agreement between the journal Editor in Chief, Professor Keith Harding; and John Wiley & Sons Ltd. Following an investigation by the publisher, all parties have concluded that this article was accepted solely on the basis of a compromised peer review process. The editors have therefore decided to retract the article. The authors did not respond to our notice regarding the retraction.

